# Readability of Korean-Language COVID-19 Information from the South Korean National COVID-19 Portal Intended for the General Public: Cross-sectional Infodemiology Study

**DOI:** 10.2196/30085

**Published:** 2022-03-03

**Authors:** Hana Moon, Geon Ho Lee, Yoon Jeong Cho

**Affiliations:** 1 Department of Family Medicine Daegu Catholic University School of Medicine Daegu Republic of Korea

**Keywords:** COVID-19, health literacy, readability, public health, health equity, consumer health information, information dissemination, health education, eHealth, online, social media, pandemic, infodemic

## Abstract

**Background:**

The coronavirus pandemic has increased reliance on the internet as a tool for disseminating information; however, information is useful only when it can be understood. Prior research has shown that web-based health information is not always easy to understand. It is not yet known whether the Korean-language COVID-19 information from the internet is easy for the general public to understand.

**Objective:**

We aimed to evaluate the readability of Korean-language COVID-19 information intended for the general public from the national COVID-19 portal of South Korea.

**Methods:**

A total of 122 publicly available COVID-19 information documents written in Korean were obtained from the South Korean national COVID-19 portal. We determined the level of readability (at or below ninth grade, 10th to 12th grade, college, or professional) of each document using a readability tool for Korean-language text. We measured the reading time, character count, word count, sentence count, and paragraph count for each document. We also evaluated the characteristics of difficult-to-read documents to modify the readability from difficult to easy.

**Results:**

The median readability level was at a professional level; 90.2% (110/122) of the information was difficult to read. In all 4 topics, few documents were easy to read (overview: 5/12, 41.7%; prevention: 6/97, 6.2%; test: 0/5, 0%; treatment: 1/8, 12.5%; *P*=.006), with a median 11th-grade readability level for overview, a median professional readability level for prevention, and median college readability levels for test and treatment. Difficult-to-read information had the following characteristics in common: literacy style, medical jargon, and unnecessary detail.

**Conclusions:**

In all 4 topics, most of the Korean-language COVID-19 web-based information intended for the general public provided by the national COVID-19 portal of South Korea was difficult to read; the median readability levels exceeded the recommended ninth-grade level. Readability should be a key consideration in developing public health documents, which play an important role in disease prevention and health promotion.

## Introduction

Digital health literacy is the ability to seek, find, understand, and appraise health information from electronic sources, and the subsequent ability to apply the knowledge to address a health problem [1]. Many people have difficulty understanding written information worldwide, including approximately 9.6 million Koreans [2] and approximately 75 million Americans [3]. Therefore, providing health information without considering the population’s literacy level does not guarantee people’s ability to understand and apply the information [4].

Society should ensure that vulnerable individuals are not left behind. Health literacy is a social determinant of health—people with lower literacy skills are less likely to access information or health care services at the same level as those with higher literacy skills [5]. This may contribute to poor health outcomes, such as lower adherence to infection control and prevention measures, ineffective use of health care, and high mortality rates [5-7]. In this context, public health researchers and policy makers have recently extended the concept of health literacy from personal reading skills to organizational health literacy [5,8,9]. The US Department of Health and Human Services’ health strategy [10] emphasized that it is the responsibility of health care organizations to design and deliver health care services and information relating to health in an accessible and understandable format. The South Korean government also includes developing easy-to-read health information in its action plan to reduce health inequity [11]. These public health efforts to achieve health equity will reduce the risk of increasing health disparities within and between countries [10-13].

Readability refers to how easy a text is to read and understand, and it is commonly measured by school grade level (kindergarten to postgraduate school) [14]. Generally, a text is considered easy to read when written below the average reading level of an adult [14]. Health care authorities have encouraged enhancing the readability of health care information, recommending that health information intended for the public be written below a sixth-grade reading level [15-17]. The South Korean government did not establish a standard for the readability of health information despite doing so for other information documents, with the Easy-to-Understand Legislation Project in 2006, which recommended that documents for the general public be written at or below a ninth-grade reading level [18]. This standard was based on the average reading level of Korean adults and the 9 years of free compulsory education that South Koreans receive [19]. The ninth-grade level was also used as a standard for sufficient literacy skills required for daily life in the 2017 Second Korean Adult Literacy Survey conducted by the Ministry of Education and the National Institute for Continuing Education [2]; thus, similarly, this study considers public health information adequately readable when written at or below that of a ninth-grade reading level.

The COVID-19 pandemic has changed the way we communicate, with more people relying on the internet as their primary source of information [20]. COVID-19–related searches have surged and been predominant in 2020 [21,22]. Consequently, web-based communication regarding the risks of the virus has become increasingly important [23,24]. According to a UN report [25], governments have used websites to provide accurate information for the public since the early days of the COVID-19 pandemic. Although health care’s digital transformation has increased the accessibility of information [26], many people may not understand the COVID-19 information shared by health care authorities. Prior research [15,27-34] has shown that most available health information is difficult for the general public to understand. For example, a systematic review [15] of 157 cross-sectional studies concluded that the US and Canada’s web-based health information is written above the average reading level of the population that it aims to inform. Patient information leaflets written in Korean were also found to be written above the average reading level of South Korean adults [27-30], and recently, studies [31-34] have reported that web-based COVID-19 information (written in English) is considered too difficult to understand by the public. However, no studies have been conducted on Korean-language web-based COVID-19 information. We aimed to address this literature gap by evaluating the readability of Korean-language COVID-19 resources. We investigated three research questions: (1) Is Korean-language COVID-19 information provided for the general public on the national COVID-19 portal of South Korea written at or below the recommended ninth-grade level? (2) Does readability differ across topics? (3) What are the characteristics of difficult-to-read information, and how can we improve readability?

## Methods

### Search Strategy

Information posted between February 3, 2020 and February 10, 2021 was downloaded from the national COVID-19 portal of South Korea [35] on February 10, 2021. Any subsidiary webpages or subdirectories that had information accessible by the public were also assessed using software (Sitechecker, version February 2021; Boosta Inc). All documents were initially screened by title and the main text was reviewed by 2 authors (HM and GHL) independently, using inclusion and exclusion criteria. Any discrepancies were resolved through discussion with the third author (YJC). Information that contained (1) COVID-19 information intended for the general public, (2) was written in Korean-language, and (3) provided by the South Korean government was included. Information that was (1) in other languages, (2) noneducational (such as press releases or daily case updates), (3) not in a written format (ie, videos and images), or (4) intended for public health and health care professionals was excluded. We also excluded duplicate documents.

### Ethics

In this study, there were no human participants or assigned interventions; therefore, we did not seek specific ethical approval from an institution review board.

### Topic Classification

Included documents were classified by topic—overview, prevention, test, or treatment—by 2 of the authors (HM and GHL) independently, and any disagreement was resolved via discussion with the third author (YJC). The *overview* category included documents about COVID-19 risk factors, transmission, and the natural course of the disease. The *prevention* category included documents discussing cleaning, disinfection, physical distancing, personal protective equipment, and vaccination. The *test* category included documents discussing indications, screening and confirmation tests, or the interpretation of test results. The *treatment* category included documents about self-care and patient care.

### Text Preparation

Documents were formatted as raw text files using Notepad (Microsoft Inc). Any text not directly related to public education was deleted, such as date, author information, titles, figures, tables, legends, references, and copyright information [31,33,36].

### Readability Assessment

We assessed text readability using a tool for Korean text (Natmal, version 2019; Lexical data-processing research institute) that determines the readability level based on sentence length and word difficulty [37]. The Natmal database contains 500,000 words classified into 9 difficulty levels by grade [38]. The frequency of words used in the text is measured for each grade and then weighted according to the number of words listed in the database [37]. We grouped text into 4 levels: professional, college, 10th to 12th grade, and at or below ninth grade. Difficult-to-read information was defined as information with a readability level exceeding the ninth-grade level (professional, college, 10th to 12th grade), and easy-to-read information was defined as information with a readability level at or below the ninth-grade level [2].

### Enhancing Readability

We analyzed the characteristics of difficult-to-read information to determine common characteristics. Out of the documents that were written at a professional level, 3 documents, each representing a characteristic, were selected after discussion among the authors. To modify the readability level, we addressed each problem characteristic. The readability of each revised document was reassessed with the tool.

### Statistical Analysis

All analyses were conducted with the R statistical software (version 3.6.3; R Foundation for Statistical Computing). Results were considered significant with *P*<.05. Shapiro-Wilk tests were used to assess the data normality. The categorical variable (readability level) was presented as frequency and percentage, and continuous variables (reading time, character count, word count, sentence count, and paragraph count) were presented as median and interquartile range. Chi-square tests or Fisher exact tests were used to calculate *P* values for categorical variables. Kruskal-Wallis tests were used to calculate *P* values for continuous variables.

## Results

### Readability of the Documents

**Figure 1:**
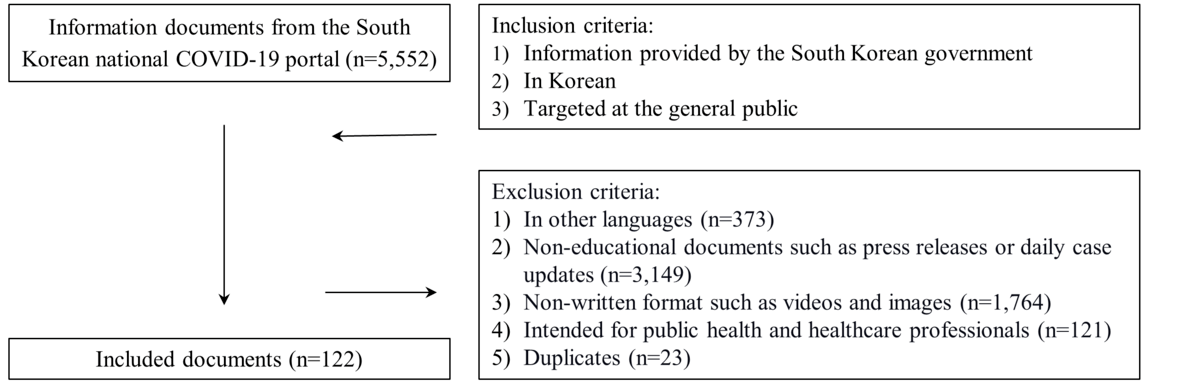
Data collection flowchart.

A total of 122 educational documents were included in this study (Figure 1). The median readability level was *professional*; 9.8% (12/122) of documents were classified as easy to read, and 90.2% (110/122) were classified as difficult to read (Table 1).

**Table 1:** Readability characteristics by level.

Characteristic	All (n=122)	Level					*P* value
		Professional (n=66)	College (n=33)	10th to 12th grade (n=11)	At or below ninth grade (n=12)		
Reading time (seconds), median (IQR)	78.4 (43.0, 161.3)	129.4 (57.2, 191.6)	64.4 (43.9, 107.7)	38.9 (33.4, 93.9)	32.8 (24.9, 48.8)	<.001	
Character count, median (IQR)	1350.5 (703.0, 2418.0)	2003.0 (945.0, 2926.0)	1242.0 (739.0, 1837.0)	701.0 (620.5, 1609.0)	632.5 (475.5, 878.0)	<.001	
Word count, median (IQR)	264.5 (145.0, 544.0)	436.5 (193.0, 646.0)	217.0 (148.0, 363.0)	131.0 (112.5, 316.5)	110.5 (84.0, 164.5)	<.001	
Sentence count, median (IQR)	31.5 (18.0, 60.0)	47.0 (24.0, 68.0)	26.0 (14.0, 36.0)	21.0 (17.5, 36.0)	17.0 (10.0, 21.5)	<.001	
Paragraph count, median (IQR)	35.5 (20.0, 84.0)	75.5 (35.0, 121.0)	27.0 (17.0, 41.0)	23.0 (19.5, 33.0)	13.5 (9.5, 17.0)	<.001	

### Readability Among the Documents of Different Topics

Included documents did not evenly cover the 4 topics (Table 2), with most (97/122, 79.5%) covering the topic *prevention*. For all topics, median readability was classified as difficult (overview: 11th-grade level; prevention: professional level; test: college level; treatment: college level), and there were few (all cases: *P*=.006) easy-to-read documents (overview: 5/12, 41.7%; prevention: 6/97, 6.2%; test: 0/5, 0%; treatment: 1/8, 12.5%).

**Table 2:** Readability among the documents of different topics.

Characteristic		All (n=122)	Topic				*P* value
			Overview (n=12)	Prevention (n=97)	Test (n=5)	Treatment (n=8)	
Readability level, median		Professional	11th grade	Professional	College	College	—^a^
**Readability level, n (%)**							.006
	Professional	66 (54.1)	3 (25.0)	59 (60.8)	2 (40.0)	2 (25.0)	
	College	33 (27.0)	3 (25.0)	23 (23.7)	3 (60.0)	4 (50.0)	
	10th to 12th grade	11 (9.0)	1 (8.3)	9 (9.3)	0 (0.0)	1 (12.5)	
	At or below ninth grade	12 (9.8)	5 (41.7)	6 (6.2)	0 (0.0)	1 (12.5)	
Reading time (seconds), median (IQR)		78.4 (43.0, 161.3)	35.4 (27.4, 57.4)	97.6 (49.8, 170.5)	56.9 (32.0, 64.4)	42.1 (29.9, 93.8)	<.001
Character count, median (IQR)		1350.5 (703.0, 2418.0)	663.0 (565.5, 1176.0)	1559.0 (830.0, 2544.0)	1049.0 (609.0, 1331.0)	763.5 (571.5, 1556.5)	.01
Word count, median (IQR)		264.5 (145.0, 544.0)	119.5 (92.5, 193.5)	329.0 (168.0, 575.0)	192.0 (108.0, 217.0)	142.0 (101.0, 316.5)	<.001
Sentence count, median (IQR)		31.5 (18.0, 60.0)	16.0 (10.0, 27.0)	37.0 (20.0, 63.0)	19.0 (11.0, 29.0)	15.0 (11.0, 29.0)	<.001
Paragraph count, median (IQR)		35.5 (20.0, 84.0)	14.0 (10.5, 30.0)	44.0 (23.0, 97.0)	26.0 (16.0, 31.0)	18.5 (11.5, 36.5)	<.001

^a^This comparison was not made.

### Enhancing Readability

Difficult-to-read information had 3 characteristics in common. They were written in literacy style, with medical jargon, and with unnecessary detail (Table 3).

Document A was the answer to the question, “Will I catch COVID-19 if I travel on a bus or subway train previously used by a confirmed patient?” It was written in literacy style, making it difficult to read. It was changed from literacy style to colloquial to improve its readability.

Document B was the answer to the question, “What are the symptoms of COVID-19?” The original version described the symptoms at length, making it difficult to identify important information. To make the document easier to understand, we included only typical symptoms for each organ rather than all possible symptoms. The medical jargon used in document B was replaced with common words to improve its readability. Medical terms, such as dyspnea, hemoptysis, emesis, anosmia, and ageusia, were replaced with commonly used words, such as shortness of breath, coughing up blood, vomiting, and loss of smell or taste.

Document C was the answer to the question, “How is the test for COVID-19 done?” The original version used medical jargon and included excessive detail in the explanation. To improve its readability, the content was summarized, and the unnecessarily detailed information included in document C was replaced with information tailored to the general public.

**Table 3:** Original text [35] and text with revisions to enhance readability.

Document	Original version	Suggested revision
A, literacy style	When riding on public transportation such as buses or subways, if you touch a handle that has been contaminated by the coronavirus, you could become infected with the virus through your eyes, nose, and mouth. However, proper wearing of a mask and hand sanitization can lower the risk of COVID-19 infection.	You can catch the virus through your eyes, nose, or mouth after touching a handle that has the virus on it. However, if you wear a mask and wash your hands, you can lower your chances of contracting COVID-19.
B, medical jargon	Symptoms such as a fever of 37.5 ℃ or higher, cough, dyspnea, chills, muscle pain, headache, sore throat, anosmia, ageusia, pneumonia, fatigue, decreased appetite, phlegm, confusion, dizziness, and a runny or stuffy nose can indicate COVID-19. Hemoptysis, chest pain, conjunctivitis, skin symptoms, or digestive system symptoms such as nausea, emesis, and diarrhea may also indicate COVID-19.	Fever and cough are common. Having COVID-19 can make you feel cold and tired, and you may have difficulty in breathing. You may lose your appetite and have a sore throat, body aches, nausea, or diarrhea. You may not have any sense of smell or taste. Some people may not have any symptoms and feel normal, but they can still spread the virus.
C, unnecessary detail	Samples are collected by physicians, nurses, and medical technicians at designated locations. Upper respiratory tract sampling is mandatory, while lower respiratory tract sampling is optional for patients with sputum. You may experience discomfort or pain while the sample is being collected. The upper respiratory tract sample is a combination of a nasopharyngeal and oropharyngeal swab in one tube. A nasopharyngeal swab involves inserting a cotton swab into the nostril until it reaches the posterior nares. An oropharyngeal swab includes inserting a cotton swab to scrape the inside of the throat. A lower respiratory tract sample is collected by spitting sputum into a container, ensuring that it is not contaminated by other liquids.	You can get tested at screening centers or drive-through facilities. There is a swab and a spit test. In the swab test, a doctor or nurse inserts a cotton swab into your nose or throat. You may feel some discomfort. For the spit test, you are asked to spit thick mucus from your throat into a tube. You will receive your test results through text message.

## Discussion

### Principal Results and Study Strengths

This study shows that 90.2% of the information available to the public was difficult to read. Of the documents discussing the prevention of COVID-19, only 6.2% (6/97) could be rated as easy to read. This is noteworthy as it shows that very few documents would effectively be able to spread prevention knowledge to the general population. To encourage people to adopt personal protective measures, such as wearing masks and washing hands during the pandemic, the government should prioritize making information on prevention easier to understand. Moreover, easy-to-read documents that were available did not cover all relevant topics equally, such as overview (n=5), prevention (n=6), test (n=0), and treatment (n=1). To make sure that those with lower literacy skills have access to information on public health and safety topics at the same level as people with higher literacy skills.

To the best of our knowledge, this is the first study to evaluate the readability of web-based COVID-19 information written in Korean. Korean is the 20th most spoken language globally, with approximately 82 million speakers [39]. Moreover, South Korea has a large population of older individuals; in 2020, adults aged 65 years and older accounted for 15.7% of the population [40], and it is estimated that South Korea will become a super-aged society in 2025, when the proportion of older adults is expected to reach 20.3% [40]. The proportion is likely to increase to 43.9% by 2060 [40]. Older adults are an important target group when assembling easy-to-understand COVID-19 information because they are at high risk for developing serious complications from COVID-19 [41]. In addition, many older adults have low literacy skills [42]. For example, 71% of Americans older than 60 years were reported to have difficulties understanding written information [3]. Nearly one-third (31.27%) of Korean adults aged 55 to 65 years could read the words but could not understand sentences or long texts [19]. Older adults with lower literacy skills are more likely to be marginalized by the government’s health care or welfare system because they cannot follow instructions for filling out forms.

The documents used in this study, from the national COVID-19 portal run by the government, which is an integrated communication channel that compiles all the COVID-19 information created by various government agencies [35], are likely an accurate reflection of the information currently accessible to the public. The Korean government’s message was amplified through social media platforms (such as Twitter and Facebook) and traditional media (TV and newspapers), thus reaching every corner of the country [43].

### Advantages and Disadvantages of the National COVID-19 Portal of South Korea for Distributing Information

The national COVID-19 portal of South Korea has several advantages for disseminating information. First, the website is highly accessible; it appears at the top of the first search results page when searching for COVID-19 on 3 major local search platforms: Naver, which has a 68.9% search engine market share in South Korea; Google, which has a 21.4% market share; and Daum, which has a 7.5% market share [44]. This national COVID-19 portal ranked first in web traffic among South Korean government websites [45], and the website ranked 10th in global rankings for health conditions and concerns [46]. Most health information seekers begin their search activities with search engines [47]; therefore, it is highly likely that Koreans who searched for COVID-19 information on the internet visited the national COVID-19 portal of South Korea. Furthermore, the information on the national COVID-19 portal are convenient for users because they can access any page without logging in, the documents can be downloaded in various formats, and anyone may freely use these documents for public purposes without any copyright restrictions.

Although the national South Korean COVID-19 portal may serve as a key communication platform between health care authorities and the public, it does not have a user-friendly interface. The web pages of the portal are not divided according to the target audience. As a result, medical professionals visiting this website may waste time reading superficial information, and users who are not medical experts may be overwhelmed with unnecessarily detailed explanations. By separating the pages or sections according to audience type (health care workers, the general public, people with low literacy skills), users may then have a more convenient way to access user-friendly information.

### Readability of Web-Based COVID-19 Information Generated by Other Public Health Agencies

The websites of the US Centers for Disease Control and Prevention and the National Health Service England have distinct sections for health care workers, the general public, and people with low literacy skills [48-51]. The web page for health care professionals provides COVID-19 training, such as clinical guides for managing cancer patients and patients requiring endoscopy during the COVID-19 pandemic [50]. Their easy-to-read sections provide information on COVID-19 basics, such as advice surrounding staying at home, COVID-19 vaccine during pregnancy, and what to expect after receiving a COVID-19 vaccine [51].

However, recent studies [34,52,53] have shown that some public health agencies have also failed to provide information in an easy-to-read form. Valizadeh-Haghi et al [52] examined the readability of English-language COVID-19 information based on website categories (ie, news, governmental, commercial, organization, educational) and concluded that that the readability levels in all categories exceeded the recommended level and that commercial websites had better readability than governmental websites. Mishra et al [34] reported that the readability of English-language COVID-19 information on 18 government and international public health agency websites did not meet the recommended readability level. Halboub et al [53] investigated 36 Arabic-language websites on COVID-19 and reported that 66.7% of the included websites were easy for the general public to read. Kruse et al [33] conducted a study of COVID-19 information provided by 8 US academic medical centers and reported that 0.7% of information was written at or below the sixth-grade level.

### Comparison With Literature on Readability of Outbreak-Related Information

Reading outbreak-related information poses extra challenges to a layperson because of the use of medical jargon [54-56]. Unfamiliar terms, such as *waterborne*, *vertical*, *zoonotic infection*, *herd immunity*, *incubation period*, *cohort isolation*, *outbreak*, *epidemic*, and *pandemic*, are frequently used. Previous studies [57,58] have shown that information on infectious diseases had poor readability; for example, Ebola virus–related information provided by public health agencies was written at readability levels higher than recommended [57], and Basch et al [58] reported that 93% of web-based Zika virus–related information was difficult to read.

Moreover, understanding COVID-19 information is more challenging because of the use of new words and phrases, such as *social distancing*, *un-tact* (noncontact), *new normal*, and *covideo party*. COVID-19 search results are also difficult for the public to understand—only 17.2% of COVID-19–related web pages were at a readable level [32], and Google-searched information regarding COVID-19 (0/150 articles) did not meet recommended readability levels.

Recent readability studies on English texts have been conducted on various types of texts on specific topics such as those used with vaccine clinical trials, privacy policies, and tests. For example, Emanuel and Boyle [59] reported that informed consent texts for COVID-19 vaccine trials were long and difficult to read. Zhang et al [60] reported that explanations of privacy policies of COVID-19 contact-tracing apps were written at readability levels higher than those recommended. Garcia et al [61] investigated the readability of web-based information on COVID-19 testing and reported that only 6 of 50 websites had appropriate readability.

Interestingly, Mishra et al [34] included information written in English posted on the South Korean government website and reported that it was written above the 11th-grade level; however, these results may not truly represent COVID-19 information commonly shared by Koreans, as South Korea is not an English-speaking country. Korean is the only official language of South Korea; paperwork and internet activities in this region are mainly in Korean. Therefore, information in Korean, rather than information in English, should be analyzed to yield results that represent COVID-19 information commonly shared by Korean.

### Implications for Practice

To improve the readability of COVID-19 information aimed at the general public, we urge the South Korean government to introduce the following measures. First, the national COVID-19 portal should be organized according to audience type (ie, health care workers, the general public, and those with a low level of education) to optimize the user experience of each type of audience. Second, guidelines on how to draft easy-to-understand health information for Korean speakers should be developed using plain-writing guidelines that include the principles and skills for easy-to-read writing—the target audience should be identified, important points should be prioritized, information should be provided step by step, foreign words should be reduced, short sentences should be used, important topics should be summarized at the end [62],and synonyms should be used to replace medical jargon with everyday words [63,64].

### Limitations

Our study has several limitations. First, the results might be biased since only one tool was used to assess readability. Second, our results are representative only of the search time frame. Numerous COVID-19 studies have been conducted, and web-based information related to it is constantly changing [65]. Third, there is no verifiable information regarding who has accessed or read what information in the study because collecting or using any personal information was not allowed. Fourth, we only used the readability tool and did not test any information with actual reader. Fifth, we only analyzed text and did not consider other factors that could affect readers' understanding, such as layout, figures, or videos.

### Future Directions

Future research should investigate the impact of nontext elements (ie, figures, infographics, videos) on information comprehension. In regions where two or more languages are spoken, it is also necessary to assess the readability of COVID-19 information in other languages. and differences in readability between languages should be assessed.

### Conclusions

Readability levels of COVID-19 web-based information provided by the national COVID-19 portal of South Korea exceeded the recommended ninth-grade level. Efforts are needed to provide easy-to-read information to reach more people during a public health crisis. We hope that this study serves as a call to action for health care authorities to develop better guidelines that encourage an easy-to-read format so that information is provided at a level that most readers can understand and apply.
